# Metformin in Lung Cancer: Review of in Vitro and in Vivo Animal Studies

**DOI:** 10.3390/cancers9050045

**Published:** 2017-05-06

**Authors:** Michael Yousef, Evangelia Tsiani

**Affiliations:** 1 Department of Health Sciences, Brock University, St. Catharines, ON L2S 3A1, Canada; my11dq@brocku.ca; 2 Centre for Bone and Muscle Health, Brock University, St. Catharines, ON L2S 3A1, Canada

**Keywords:** metformin, lung cancer, cell signaling, proliferation

## Abstract

Cancer cells display enhanced growth rates and a resistance to apoptosis. The ability of cancer cells to evade homeostasis and proliferate uncontrollably while avoiding programmed cell death/apoptosis is acquired through mutations to key signaling molecules, which regulate pathways involved in cell proliferation and survival and these mutations allow them to develop resistance to many chemotherapeutic agents, highlighting the need for development of new potent anti-cancer agents. Metformin has long been used as a treatment for type 2 diabetes and has recently attracted attention as a potential agent to be used in the treatment of cancer. The present review summarizes the existing in vitro and in vivo animal studies focusing on the anti-lung cancer effects of metformin and its effects on key proliferative and anti-apoptotic signaling pathways.

## 1. Introduction

Cancer cells are characterized by their high rate of proliferation and resistance to apoptotic cell death [1]. There were 1.56 million deaths globally due to lung cancer in 2012, representing approximately 19.4% of all cancer related deaths [2]. It was estimated that, in 2016, new lung cancer cases will account for 14% of all new cancer cases and lung cancer related deaths will account for 26% of all cancer related deaths [3].

Most cancers that originate in the lungs are known as carcinomas, and are subdivided into non-small-cell lung cancer (NSCLC) and small-cell lung cancer (SCLC). NSCLC accounts for approximately 85% of all lung cancer cases [3]. There are three subtypes of NSCLC: adenocarcinoma, squamous cell carcinoma and large cell carcinoma. Adenocarcinoma is the most common type, accounting for approximately 40% of all lung cancer cases and is most often characterized by significant production of mucus [4]. Squamous cell carcinoma is more closely related to tobacco smoking than any other type of lung cancer and often occurs centrally in larger bronchi [4,5]. Large cell carcinoma is a heterogeneous group of undifferentiated malignant neoplasms that lack features of other types of lung cancer [4]. SCLC accounts for approximately 15% of all lung cancer cases [3]. Typically, SCLC presents in the central airways and attacks the submucosa layer leading to narrowing of the airways [5]. Compared to NSCLC, SCLC has a shorter doubling time and metastasizes earlier. In fact, over 70% of SCLC patients present with metastatic disease [5].

Cancer cells exhibit various mutations that increase the expression/activation of growth factor (GF) receptor proteins such as epidermal growth factor receptor (EGFR) [6,7,8,9]. Growth factors bind to their receptors, receptor auto-phosphorylation occurs leading to activation of downstream signaling cascades such as the phosphoinositide-3-kinase (PI3K)/Protein kinase B (Akt)/mammalian target of rapamycin (mTOR) pathway that results in increased survival/proliferation and inhibition of apoptosis [10,11,12,13]. Another pathway activated by GF signaling is the RAS/mitogen activated protein kinases (MAPK) pathway. GF binding to the activated receptor through the involvement of the Grb2/SOS complex renders Ras in the GTP-bound form, activating Raf, MEK and MAPK further downstream [13,14,15,16]. Cancer cells often exhibit mutations that allow them to constitutively enhance proliferation while inhibiting apoptosis/cell death pathways.

Lung cancer is treated with surgery, radiation therapy, chemotherapy, specific and targeted therapy or a combination of these different approaches. Due to a high level of metastasis and post-surgical relapse, chemotherapy has become the most prevalent form of treatment. Unfortunately, cancer cells in general and lung cancer specifically are characterized by mutations and over activation of the PI3K-Akt-mTOR [17,18] and the Ras-MAPK [14,19] cascades that allow them to develop resistance to many first line chemotherapeutic agents highlighting the need to develop new chemotherapeutic agents.

Metformin was first used in medieval Europe, where it was extracted from *Galega officinalis* also known as the French lilac and it was used to treat polyuria. Metformin is now used as a first-line medication for the treatment of type 2 diabetes mellitus. Research interest on the anticancer effects of metformin was initiated in 2005 in a study by Evans et al., showing reduced risk of cancer in type 2 diabetes patients receiving metformin [20]. This study sparked scientific interest on the anticancer effects of metformin. Further studies examining the anticancer effects of metformin have been performed and many other clinical trials are ongoing [21,22]. For more information on the ongoing clinical trials, the reader can see the following websites https://www.clinicaltrials.gov and https://health-products.canada.ca/ctdb-bdec/index-eng.jsp.

Many studies have been performed using metformin in different cancer cell lines and in in vivo animal models of cancer [23,24,25,26,27,28,29,30,31,32,33]. In the present review, we summarize all in vitro and in vivo animal studies examining the effects of metformin in lung cancer. To establish a systematic literature review, we used the online search engine PubMed. We searched the key phrases: “Metformin” and “Lung Cancer”. All published studies pertaining to our topic were included in the current review. In vitro and in vivo studies on the effects of metformin have been summarized in separate sections and sorted by histology and treatment approach (single agent or combination therapy) resulting in a clear, detailed and inclusive summary of the existing literature. This review also focuses on the mechanistic data provided by these studies, which will be beneficial for future research to help focus efforts on identifying the main mechanisms involved in the anticancer action of metformin. The studies presented in the text are also summarized, organized and presented in a table format to allow the reader to extract the information easily. This is a comprehensive systematic review and adds to the existing literature by summarizing all relevant in vitro and in vivo animal studies using metformin in lung cancer.

## 2. Effects of Metformin in Lung Cancer: In Vitro Studies

Metformin has been used as a single agent in many different lung cancer cells, and the data obtained indicate numerous anticancer properties (Table 1). Exposure of A549, RERF-LC-A1, IA-5 and WA-hT cells to metformin (1–20 mM) resulted in a significant decrease in cell proliferation, significant induction of apoptosis as well as decreased colony formation accompanied by a significant induction of G_0_/G_1_ cell cycle arrest [34]. Treatment of Calu-1 and Calu-6 cells with metformin (0.3–5 mM) was shown to decrease proliferation in Calu-6 cells which express lower levels of Calveolin-1. This was associated with inhibition of IGF-1 dependent phosphorylation (activation) of the proto-oncogene Akt [35]. FOXO3a, a transcription factor involved in initiation of apoptosis, is phosphorylated by Akt resulting in its nuclear exit and therefore inhibition of apoptosis. IGF-1 dependent FOXO3a phosphorylation and nuclear exit was shown to be inhibited by metformin. Furthermore, it was observed that phosphorylation/activation of the energy sensor AMPK was increased by metformin, an effect that required the involvement of calveolin-1 [35]. Do et al., 2013 treated A549 cells with metformin (1–10 mM) and found that heme oxygenase (HO-1) mRNA and protein levels were suppressed indicating decreased levels of oxidative stress, Nrf2, a transcription factor that regulates production of antioxidant proteins that protect against oxidative damage, was reduced [36]. Additionally, Raf/ERK phosphorylation was also suppressed [36]. Exposure of PC9 cells to metformin (1–32 mM) resulted in an inhibition of proliferation [37]. A study by Ko et al., 2013 found that exposure of A549 and H1975 cells to metformin (5–50 μM) resulted in increased cytotoxicity and decreased thymidine phosphorylase (TP) and excision repair cross-complementation 1 (ERCC1) mRNA and protein levels, both of which are associated with repairing DNA breaks [38]. Interestingly, a significant decrease in MEK/ERK phosphorylation was seen with metformin treatment [38]. Exposure of A549 and A431 cells to metformin (1–10 mM) was found to induce apoptosis and inhibit proliferation as well as reduce Akt and mTOR phosphorylation [39]. Treatment of H1299, GLC82, H1975, CALU-3, CALU-3 GEF-R, H460 and A549 cells with metformin (0.1–20 mM) resulted in inhibition of proliferation and induction of apoptosis [40]. Metformin treatment alone was also found to activate MAPK [40], while in another study in squamous lung cancer cells [41] metformin inhibited MAPK. Interestingly, increased activation of MAPK in breast cancer [42] and adrenal gland cancer [43] was associated with a decrease in cell proliferation and an increase in apoptosis indicating that MAPK activation in specific cells can lead to apoptosis. Exposure of Calu-1 cells to metformin (0.0375–10 mM) resulted in a significant induction of apoptosis which was associated with decreased glucose uptake marked by an inhibition of the key glycolytic enzyme hexokinase-II [44]. These data provide strong evidence that metformin had an inhibitory effect on cancer cell glycolysis, counteracting the Warburg effect [45,46]. A549 cells treated with metformin (0.5–8 mM showed inhibition of proliferation and induction of apoptosis which was associated with increased p38 MAPK phosphorylation [47]. In another study, A549 cells treated with metformin (10 mM) were shown to have decreased expression of B-lymphoma Moloney murine leukemia virus insertion region-1 (Bmi-1). Bmi-1 is an oncogene that alters the cell cycle and apoptosis by promoting tumor cell self-renewal and epithelial to mesenchymal transition (EMT). Metformin’s inhibition of EMT has also been reported in several other studies, Kurimoto et al. reported that both PC-9 and HCC-827 adenocarcinoma cells treated with TGF-β and FGF-2 to induce EMT, had elevated levels of PD-L1 (a marker of EMT) and increased resistance to gefitinib and cisplatin, however treatment with metformin (0.1–10 mM) suppressed PD-L1 expression and reversed resistance to gefitinib and cisplatin [48]. Cufi et al. also reported that treatment of MCF-7 breast cancer cells with metformin impeded the TGF-β induced EMT and increased the levels of the epithelial marker E-cadherin [49]. In addition metformin prevented the TGF-β induced cell scattering and accumulation of the mesenchymal marker vimentin in Madin-Darby canine kidney (MDCK) cells) [49]. These metformin effects may be mediated by an increase of miR-15a, miR-128, miR-192 and miR-194 which inhibit Bmi-1 mRNA translation [50]. A study by Li et al., 2015 found that treatment of PC-9 and PC-9GR cells with metformin (5 mM) resulted in decreased markers of pulmonary fibrosis associated with decreased TGF-β activation and reduced levels of the downstream signaling molecules COL1A1, pSMAD2, pSMAD3, pSTAT3, pAKT and pERK [51]. Treatment of H522, H2342, H2405, A549, SPC-A-1, SW900, H1869, SK-MES-1, H661, H1299 and H1281 with metformin (1.25–5 mM) resulted in decreased proliferation and decreased stem cell marker levels [52]. Metformin was also shown to decrease nemo-like kinase (NLK), a member of the MAPK pathway, Nanog, a transcription factor involved in self-renewal of undifferentiated embryonic stem cells as well as the levels of the transcription factors c-Myc and KLF4 [52]. Treatment of A549 cells with metformin (5–50 mM) resulted in increased apoptosis and G_0_/G_1_ cell cycle arrest, decreased Bcl-2 and increased Bax protein levels [53]. Exposure of H460 and H1299 cells to metformin (5–20 mM) was shown to inhibit proliferation, induce apoptosis, induce G_0_/G_1_ cell cycle arrest, increase AMPK phosphorylation and suppress total and phosphorylated mTOR and p70S6K levels [54].

## 3. Effects of Combined Metformin Treatment in Lung Cancer: In Vitro Studies

Apart from all the above-mentioned studies where metformin was used as a monotherapy, more recent studies have examined the effect of metformin treatment in combination with other agents/treatment strategies (Table 2). Treatment of H520 and H1703 cells with 0.1–1 mM metformin in combination with 1–10 μM gefitinib, an EGFR inhibitor, was found to increase gefitinibs’ cytotoxic effect. Metformin potentiated the growth inhibitory effect of gefitinib by decreasing the gefitinib induced MSH2 expression [41]. Furthermore, treatment of PC-9, PC-9GR and H1650-M3 cells with 5 mM metformin and 1–16 μM gefitinib or 2.5–20 μM erlotinib, another EGFR inhibitor, was shown to re-sensitize EGFR-tyrosine kinase inhibitor (TKI) resistant cells and reverse EMT through increased E-cadherin and decreased vimentin and SNAIL expression [55]. In another study H2228 and H3122 cells treated with 5 mM metformin and 400 nM crizotinib, an anaplastic lymphoma kinase (ALK) and c-ros oncogene 1 (ROS1) inhibitor, had increased crizotinib sensitivity, decreased cell proliferation and tumor invasion and increased apoptosis [56]. Additionally, metformin reversed resistance to crizotinib [50]. These effects were accompanied by decreased IGF1-R signaling and a reduction in the IGF-1 induced phosphorylation of mTOR and p70S6K [19]. Treatment with 10 mM metformin and 30 nM trametinib, a MEK inhibitor, was found to be effective in N-RAS mutant lung carcinomas, specifically SW1271 and H2347 cells. This combination was shown to decrease cell viability and reduce activity of the MAPK and PI3K/Akt/mTOR signaling pathways [57]. A549 and H460 cells treated with 0.25–2 mM metformin and 0.25–6 μM sorafenib, a tyrosine kinase inhibitor, resulted in decreased cell proliferation and an increase in AMPK activation [58]. Metformin (2 mM) combined with selumetitnib (0.01–1 μM), a MEK inhibitor, increased apoptosis and decreased proliferation of H358, Calu-3, H1299 and H1975 cells [59]. Furthermore, metformin down regulated GLI1 transcriptional activity as well as decrease the production of MMP-2, MMP-9 and NF-κB [59]. Treatment of AS2 cells with 2.5 μM–2.5 mM metformin and 2.4 μM cisplatin, a platinum based drug that interferes with DNA replication, enhanced cisplatin-induced cytotoxicity, inhibited cisplatin-induced ROS production as well as decreased secretion of VEGF [60]. Treatment of H460 cells with 15.145–60.58 mM metformin and 0.0995–0.199 mM cisplatin or 0.0926–0.1852 mM etoposide, an inducer of DNA strand breaks, decreased proliferation through reduced metabolic viability of these cells [61]. Treatment of H1650 and H1703 cells with 0.1–1 mM metformin and 0.1–1 μM paclitaxel, a drug that targets tubulin, was shown to enhance paclitaxels’ cytotoxic effects, decrease DNA excision repair protein (ERCC1) expression and p38 MAPK phosphorylation [62]. In H1299 and H1650 cells treated with 5 mM metformin and 20 μM ciglitazone, a selective PPARγ ligand, an induction of apoptosis, inhibition of growth and reduced PDK1 expression was seen [63]. In HCC4006, NCI-H1975, HCC95, NCI-H2122 and NCI-H3122 cells treatment with 0.01–10 mM metformin in combination with 5 μM salinomycin, a drug with high antimicrobial activity, was shown to promote cell death through inhibition of EGFR, Akt, mTOR and p70S6K signaling [64]. Treatment of A549 and HCC4006 cells with 1–10 mM metformin in combination with 0.1–1 μM salinomycin inhibited the TGF-β induced EMT as well as inhibited cell migration through increased E-cadherin expression [65]. A549, SK-MES-1, H520, SPC-A-1, H1975 and PC-9 cells treated with 3 mM metformin and 100 ng/mL Figitumumab, an antibody against IGF-1R, had reduced PI3K/Akt and MEK/ERK signaling cascade activation [66]. Treatment of A549 cells with 1–4 mM metformin alone or in combination with 200 ng/mL TRAIL protein enhanced TRAIL-mediated tumor cell death and decreased p62 protein levels [67]. A549 and PC9 cells treated with 20 μM metformin in combination with 20 μg/mL β-elemene had reduced growth [68]. It was observed that metformin augments the effects of β-elemene by blockage of Akt signaling and inhibition of DNMT1 expression [68]. In A549, H1299 and SK-MES1 cells treatment with 2.5 μM–5 mM metformin resulted in enhanced response to radiation treatment as was seen by enhanced inhibition of proliferation, induction of cell cycle arrest and induction of apoptosis [69].These effects were associated with activation of the AMPK-p53 pathway, inhibition of Akt and mTOR [69].

## 4. Effects of Metformin in Lung Cancer: In Vivo Studies

Metformin has not only been studied as a single agent or as part of a combination treatment in vitro, but has also been studied as both a single agent and as part of a combination treatment in animals (Table 3). In a study where Balb/c mice were injected with A549 or A431 cells then treated with 250 mg/kg/day metformin for 21 days, metformin was found to inhibit growth of K-ras mutant tumors, decreased Akt and mTOR total and phosphorylation levels and induced apoptosis [39]. Mice treated with 5 mg/mL metformin were found to have decreased lung tumorigenesis (tobacco carcinogen, NNK-induced) that was associated with a significant reduction in plasma IGF-1 levels and decreased IGF-1 signaling [70]. Additionally, phosphorylation of multiple RTKs (including EGFR, insulin receptor and VEGF) and GTP-bound Ras levels were decreased and these effects were independent of AMPK [70]. Another study found that nude mice treated with 250 mg/kg/day metformin two days before inoculation with HCC827-pSB388 cells showed decreased tumor growth, reversed IL-6 induced EMT and blocked STAT3 phosphorylation [71]. Morgillo et al., 2013 found that Balb/c mice injected with H1299 or Calu-3 GEF-R cells treated with 200 mg/mL metformin and 150 mg/kg/day gefitinib for 35 days had decreased proliferation and increased apoptosis as well as decreased EGFR phosphorylation and activation of MAPK [40]. Treatment of Balb/c mice injected with PC-9 or PC-9GR cells with 1 mg/mL metformin and 250 mg/L gefitinib for 30 days was found to block tumor growth of TKI-resistant xenografts, decrease IL-6 secretion and expression as well as reverse EMT [55].

Often excellent initial clinical responses are observed with drugs such as gefitinib, however these drugs might promote interstitial lung disease (ILD) which restricts the effectiveness of these agents in treatment. Li et al., 2015 found that treatment of Sprague Dawley (SD) rats with 300 mg/kg metformin attenuated the gefitinib-induced exacerbation of pulmonary fibrosis [51]. Metformin was also found to decrease actin, COL1A1, pSMAD2, pSMAD3, pSTAT3, p-Akt and Erk levels as well as decreased TGF-β activation and protein levels [51]. Pulmonary fibrosis, a type of ILD, has been associated with an increase in cancer risk. Balb/c mice injected with AS2 cells and treated with 500 mg/kg metformin and 4 mg/kg cisplatin showed inhibition of xenograft growth, inhibition of cisplatin-induced ROS production and inhibition of the STAT3 pathway [60]. Balb/c mice injected with A549 cells were treated with 40 mg/kg/day metformin in combination with 5 mg/kg/day cisplatin were found to have decreased tumor size mediated through decreased Bcl-2 protein levels, increased Bax protein levels and increased phosphorylation of p38 MAPK [47]. In another similar study, Balb/c mice injected with A549 cells were treated with 400 mg/kg/day metformin and 30 mg/kg/day sorafenib for 40 days and were shown to have decreased cancer cell proliferation, decreased tumor size, increased AMPK phosphorylation and inhibition of mTOR signaling [58]. Furthermore, treatment of H1299 or H1975 lung cancer cell bearing nude mice with 200 mg/mL metformin and 25 mg/kg selumetinib for 35 days was shown to inhibit EMT through increased SNAIL and decreased vimentin expression, decrease tumor metastatic behavior and decrease production of MMP-2 and MMP-9 through a reduction in NF-κB levels [59]. Balb/c mice injected with A549 or H1299 cells treated with 300 mg/kg/day metformin in combination with 10 Gy ionizing radiation (IR) until euthanasia showed inhibition of xenograft growth, activation of the AMPK-p53 pathway, inhibition of the mTOR-4EBP1 pathway, reduced angiogenesis and enhanced expression of apoptotic markers [69]. Compromised immune system responses play an important role in cancer and in recent years immune related therapies have been developed and shown to have strong clinical benefits [72,73,74]. Some of metformin’s anti-cancer effects have been proposed to be immune-mediated [75,76]. Metformin treatment resulted in rejection of solid tumors in normal but not T-cell deficient SCID mice [77], clearly demonstrating an anticancer effect via immune system modulation. Furthermore, treatment of CD8^+^ lymphocytes with low concentrations of metformin (10 μM) resulted in their protection from apoptosis, ultimately leading to enhanced anti-tumor effects [77].

## 5. Effects of Metformin in Lung Cancer: Human Studies

Although the focus of this review is on in vitro and in vivo animal studies of metformin in lung cancer, metformin has already been used in multiple clinical studies. Zhang and colleagues performed a systematic review and meta-analysis of four human studies from 2009 to 2013 and found that, in the four studies, diabetic patients receiving metformin had a significantly lowered risk of lung cancer (RR = 0.71, 95% CI: 0.55–0.95; *p* = 0.02) [78]. Studies by Hall et al. [79] and Lai et al. [80] investigated the association between diabetes and lung cancer and both reported a reduction in lung cancer risk in patients receiving metformin treatment. Specifically, Lai et al. reported that the risk of lung cancer among diabetic patients receiving metformin was 39%–45% lower [25,80]. Noto et al. performed a meta analyses of articles published before October 2011 and found the risk of lung cancer among type 2 diabetes mellitus metformin users to be 0.67 (95% CI: 0.45–0.99) [81]. Lin et al. identified 750 diabetic patients between the ages of 65–80 who were diagnosed with stage IV NSCLC between 2007 and 2009, the median survival of patients on metformin was five months compared to three months for patient not taking metformin (*p* < 0.001); the propensity score also showed that metformin use was associated with a statistically significant improvement in survival (HR = 0.80, 95% CI: 0.71–0.89) [27,82]. Tan et al. obtained data on patients with NSCLC who had diabetes from five hospitals in China between January 2004 and March 2009 and found that patients receiving chemotherapy in combination with metformin had better outcomes compared to those receiving chemotherapy in combination with insulin or other diabetic drugs, their overall survival was 20 months, 13.1 months and 13.0 months, respectively [27,83]. A study by Dhillon et al. examined 409 stage I NSCLC and diabetic patients who underwent anatomic resection from 2002 to 2011 and found that diabetes had no effect on overall survival of the patients (*p* = 0.75), but found that metformin treatment was associated with improved overall survival (*p* = 0.02) [84]. However, there are also a few studies that report no effect between metformin and risk of lung cancer. Nie et al. reported that in 11 studies found, in a systematic search up to August 2013, there was no significant association between diabetes mellitus patients receiving metformin and lung cancer risk (OR = 0.99, 95% CI: 0.87–1.12) [85]. Similarly, Franciosi et al. reported that in two randomized controlled studies (RCTs) and four observational studies, there was no significant association between metformin and lung cancer risk in RCTs and only a marginal non-significant reduction was found in observational studies in diabetes mellitus patients (OR = 0.83, 95% CI: 0.64–1.06, *p* = 0.13) [86].

In addition to the studies presented above, there are currently 15 clinical trials as of 11 April 2017 registered on the https://www.clinicaltrials.gov website that are studying the effect of metformin on lung cancer. Of the 15 currently registered studies, there are three studies examining the effects of metformin as a single agent in NSCLC. An additional four studies examining the combination of metformin and gefitinib; the combination of metformin, carboplatin, bevacizumab and paclitaxel; the effects of metformin and Nivolumab; and the combination of metformin, carbohydrate restriction and platinum based chemotherapy, aiming to examine the relationship between metformin and various chemotherapy agents in the treatment of NSCLC. There are three further studies examining the effects of combined metformin and radiotherapy treatment in NSCLC patients. One study is combining metformin with chemotherapy (paclitaxel and carboplatin) and radiotherapy. Another study is examining the effects of metformin with tyrosine kinase inhibitors (TKIs) in NSCLC patients. Additionally, a phase I study of metastatic or unresectable solid tumors is examining the combination of metformin and temsirolimus. The combination of metformin and sirolimus is also being tested in another trial in patients with advanced solid tumors. Finally one study aiming to reduce IGF-1 levels in cancer survivors is examining the effects of metformin treatment alone compared to weight loss in those survivors. Most studies involved advanced stage lung cancer, typically stage III and IV (nine studies), with the remaining studies being in stage I and II, in cancer survivors or in NSCLC patients of all stages. The typical metformin dosage in these trials is between 500 and 2000 mg daily. Sayed et al. [87] conducted a randomized controlled study on stage IV NSCLC non diabetic patients receiving gemcitabine/cisplatin alone or in combination with metformin (500 mg daily) and found that in the combination group compared to the gemcitabine/cisplatin treatment alone, the objective response rate (tumor size reduction) was 46.7% compared to 13.3% (*p* = 0.109), the overall survival was 12 months compared to 6.5 months (*p* = 0.119) and the median progression free survival was 5.5 months compared to five months (*p* = 0.062) respectively. However, there was no significant increase in toxicity in the combination group, and the percentage of patients experiencing nausea was significantly lower in the combination group (26.7%) compared to the gemcitabine/cisplatin group (66.7%) [87]. Overall, this study by Sayed et al. showed that metformin had no statistically significant improvements in objective response rate, overall survival and median progression free survival even though overall survival and median progression free survival improved slightly with metformin treatment and objective response rate improved greatly with metformin treatment, but instead metformin reduced chemotherapy induced-nausea [87].

## 6. Conclusions

In summary, all the in vitro and in vivo animal studies presented in this review indicate that metformin alone has potent anti-cancer effects and in combination studies it enhances the effectiveness of other chemotherapy agents or radiation treatment. It should be noted that the concentration of metformin used in in vitro studies are typically 1–10 mM [39,47,50,51] and in some studies as high as 50 mM [53], while the concentration of metformin in blood samples of humans treated with metformin are 2.8–15 μM [88]. We strongly urge researchers to plan future in vitro studies using metformin in the micromolar (μM) range to be physiologically/pharmacologically relevant with the in vivo levels and investigate the mechanism of action.

Although, all the in vitro and in vivo studies summarized in this review indicate potent anticancer effects of metformin, the evidence from clinical studies (meta-analysis and reviews) show both a reduced cancer risk and no response. The closed clinical trial by Sayed et al. [87] did not demonstrate significant results with metformin treatment. Currently, doubt still remains whether the anti-cancer effects of metformin observed in in vitro and in vivo studies will ultimately translate into clinical benefits in the ongoing clinical trials. While whether metformin has a clinically-relevant chemopreventive or anti-cancer effect is not clear at present, we look forward to seeing the evidence from the ongoing human clinical trial studies.

## Figures and Tables

**Table 1:** Metformin in lung cancer: in vitro studies.

Cell Type	Dose and Duration	Findings	Mechanism	Reference
RERF-LC-AI (SCC), IA-5 (LCC), WA-hT (SCLC), A549 (AC)	0.3 mM–20 mM met for 1–72 h RERF-LC-AI, IC_50_ = 6 mM A549, IC_50_ = 1 mM IA-5, IC_50_ = 5 mM WA-hT, IC_50_ = 2 mM	↓ cell proliferation ↑ apoptosis ↓ colony formation	↑ G0/G1 cell cycle arrest	[34]
Calu-1 (NSCLC), Calu-6 (AC)	0.3 mM–5 mM met for 0–72 h Calu-1 IC_50_ = 16 mM Calu-6 IC_50_ = 18 mM	↓ cell proliferation	↓ phosphorylation of IGF-IR substrates Akt and FOXO3a ↑ AMPK phosphorylation in Calu-1 cells	[35]
A549 (AC)	1–10 mM met for 24 h	↓ HO-1 mRNA and protein expression ↓ Nrf2 expression	↓ phosphorylation of Raf and ERK1/2	[36]
PC9 (AC)	1–32 mM met for 0–72 h ~ IC_50_ = 3.5 mM	↓ proliferation		[37]
A549 (AC), H1975 (AC)	5–50 μM met for 24–72 h	↑ cytotoxicity ↓ cellular TP and ERCC1 expression	↓ MEK1/2-ERK1/2 protein levels.	[38]
A549 (AC)	1–10 mM met for 24–72 h IC_50_ = 5 mM	↓ proliferation ↑ apoptosis	↓ Akt levels reducing mTOR activation	[39]
H1299 (AC), GLC82 (AC), H1975 (AC), CALU-3 (AC), CALU-3 GEF-R (AC), H460 (LCC), A549 (AC)	0.1–20 mM met for 72 h IC_50_ = 2–2.5 mM for all cells except A549 and H460 IC_50_ > 20 mM	↓ proliferation ↑ apoptosis	↑ MAPK activation	[40]
Calu-1 (NSCLC)	0.0375–10 mM met for 6, 24, 48 h	↑ apoptosis ↓ glucose uptake	↓ Hexokinase-II activity	[44]
A549 (AC)	0.5–8 mM met for 24 h ~ IC_50_ = 4 Mm	↓ proliferation ↑ apoptosis	↑ G1 cell cycle arrest ↑ p38 MAPK phosphorylation	[47]
A549 (AC)	10 mM met for 0–24 h	↓ Bmi-1 ↑ miR-15a, miR-128, miR-192 and miR-194	↑ phosphorylation of AMPK and expression of LKB1	[50]
TKI-sensitive PC-9 (AC), TKI-resistant PC-9GR (AC)	5 mM met for 48 h	↓ expression of markers of pulmonary fibrosis	↓ expression of α-actin and COL1A1 ↓ expression of pSMAD2, pSMAD3, pSTAT3, pAKT and dpERK1/2 ↓ TGF-β levels and activation.	[51]
H522 (AC), H2342 (AC), H2405 (AC), A549 (AC), SPC-A-1 (AC), SW900 (SCC), H1869 (SCC), SK-MES-1 (SCC), H661 (LCC), H1299 (AC)	1.25–5 mM met for 7 days A549 IC50 = 7.97 mM SK-MES-1 IC_50_ = 13.36 mM	↓ proliferative activities ↓ cancer stemness of A549 cells ↓ levels of stem cell markers	↓ levels of NLK, Nanog, c-Myc and KLF4	[52]
A549 (AC)	5–50 mM met for 24, 48 or 72 h 24 h, IC_50_ = 3.5 mM 48 h, IC_50_ = 8 mM 72 h, IC_50_ = 20 mM	↑ apoptosis ↑ cell cycle arrest at G_o_–G_1_ phase	↓ Bcl-2 protein levels ↑ Bax protein expression	[53]
H460 (LCC), H1299 (AC)	5, 10 or 20 mM met for 24, 48 or 72 h H460 24 h, IC_50_ > 20 mM H460 48 h, IC_50_ = 20 mM H460 72 h, IC_50_ = 10 mM H1299 24 h, IC_50_ > 20 mM H1299 48 h, IC_50_ = 20 mM H1299 72 h, IC_50_ = 20 mM	↓ proliferation ↑ apoptosis ↑ cell cycle arrest at G_o_–G_1_ phase	↑ AMPK phosphorylation ↓ mTOR and p70S6K phosphorylation	[54]

NSCLC, Non-small cell lung cancer; SCLC, Small cell lung cancer; AC, Adenocarcinoma; SCC, Squamous cell carcinoma; LCC, Large cell carcinoma; IGF-1R, Insulin-like growth factor 1 receptor; FOXO3a, Forkhead box O3; AMPK, AMP-activated protein kinase; Raf, Rapidly accelerated fibrosarcoma; ERK, Extracellular regulated kinase; ERCC1, excision repair cross-complementation 1; MEK, Mitogen-activated protein kinase kinase; Akt, Protein kinase B; mTOR, Mammalian target of rapamycin; LKB1, Liver kinase B1; COL1A1, Collagen type 1 alpha 1; SMAD2, SMAD family member 2; SMAD3, SMAD family member 3; TGF-β, Transforming growth factor β; NLK, Nemo like kinase; Nanog, Homeobox protein Nanog; KLF4, Kruppel-like factor 4; Bcl-2, B-cell lymphoma 2; Bax, Bcl-2-like protein 4; p70S6K, Ribosomal protein S6 kinase beta-1; IC_50_, half maximal inhibitory concentration.

**Table 2:** Effects of metformin in combination with other treatment in lung cancer: in vitro studies.

Cell Type	Dose and Duration	Findings	Mechanism	Reference
H520 (SCC), H1703 (SCC)	0.1–1 mM met alone or in combination with 1–10 μM gefitinib for 24 h	↑ cytotoxicity and growth inhibition by gefitinib	↓ gefitinib induced expression of MSH2 expression	[41]
PC-9 (AC), PC-9GR (AC), H1650-M3 (AC)	5 mM met alone or in combination with either 1–16 μM Gefitinib or 2.5–20 μM erlotinib for 48 h	Resensitized EGFR-TKI resistant human lung cancer cells ↓ EMT in TKI resistant human lung cancer cells	↑ E-cadherin expression ↓ Vimentin and SNAIL expression	[55]
H2228 (AC), H3122 (AC)	5 mM met alone or in combination with 400 nM crizotinib for 48 h	↓ cell proliferation ↑ apoptosis ↑ crizotinib sensitivity ↓ crizotinib resistance ↓ tumor invasion	↓ IGF1-R signaling. ↓ phosphorylation of mTOR, p70S6K and S6	[56]
SW1271 (SCLC), H2347 (AC)	10 mM met alone or in combination with 30 nM trametinib for 72 h SW1271 IC_50_ = 29.9 mM H2347 IC_50_ = 6.79 mM	Combination is effective for treatment of NRAS mutant lung carcinomas	↓ cell viability ↓ activity of MAPK and PI3K/Akt/mTOR signaling pathways.	[57]
A549 (AC), H460 (LCC)	0.5–2 mM met alone or in combination with 0.25–6 μM sorafenib, for up to 10 days	↓ proliferation	↑ AMPK activation	[58]
H358, Calu-3 (AC), H1299 (AC), H1975 (AC)	2 mM met alone or in combination with 0.01 μM or 1 μM selumetitnib for 72 h H358 IC_50_ = 1.5 mM Calu-3 IC_50_ = 1 mM H1299 IC_50_ = 1.5 mM H1975 IC_50_ = 2.5 mM	↓ proliferation ↑ apoptosis ↓ GLI1 transcriptional activity	↓ production of MMP-2 and MMP-9 by reducing NF-κB	[59]
AS2 (AC)	2.5–2.5 mM met alone or in combination with 2.5 μM cisplatin for 24–72 h	↓ secretion of VEGF ↑ cisplatin cytotoxicity ↓ cisplatin induced ROS production	↓ of STAT3 pathway	[60]
H460 (LCC)	15.145–60.58 mM met alone or in combination with 0.0995–0.199 mM cisplatin or 0.0926–0.1852 mM etoposide IC_50_ = 60.58 mM	↓ proliferation	↓ metabolic viability	[61]
H1650 (AC), H1703 (SCC)	0.1–1 mM met alone or in combination with 0.1–1 μM paclitaxel for 24 h	↑ cytotoxic effect of paclitaxel ↓ ERCC1 expression	↓ p38 MAPK phosphorylation	[62]
H1299 (AC), H1650 (AC)	5 mM met alone or in combination with 20 μM ciglitazone for 24 h	↓ growth ↑ apoptosis	↓ PDK1 expression and promoter activity	[63]
HCC4006 (AC), NCI-H1975 (AC), HCC95 (SCC), NCI-H2122 (AC), NCI-H3122 (AC)	0.01–10 mM met alone or in combination with 50–5 μM salinomycin for 48 or 72 h HCC4006 ~ IC_50_ = 2 mM H1975 ~ IC_50_ = 5 mM HCC95 ~ IC_50_ = 5 mM H2122 ~ IC_50_ > 1 mM H3122 ~ IC_50_ = 5 mM	↑ cell death	↓ EGFR signaling ↓ Akt, EGFR 1/2, mTOR and p70S6K activity	[64]
A549 (AC), HCC4006 (AC)	1–10 mM met alone or in combination with 0.1–1 μM salinomycin +10 ng/mL TGF-β for 48 h A549 ~ IC_50_ = 20 mM HCC4006, IC_50_ = 20 mM	↓ TGF-β induced EMT ↓ cell migration	↑ E-cadherin expression	[65]
A549 (AC), SPC-A-1 (AC), H1975 (AC), SK-MES-1 (SCC), H520 (SCC), PC-9 (AC)	3 mM met alone or in combination with 100 ng/mL Figitumumab for 6–48 h (Please check whether the unit missing)	↓ proliferation	↓ PI3K/Akt signaling pathways ↓ MEK/ERK signaling pathways ↓ IGF-1 receptor	[66]
A549 (AC)	1–4 mM met alone for 12 h or in combination with 200 ng/mL TRAIL protein for 2 h	↑ apoptosis	↓ c-FLIP ↓ p62 protein levels	[67]
PC9 (AC), A549 (AC),	20 μM met alone or in combination with 20 μg/mL β-elemene for 24 h	↓ cell growth	↓ Akt phosphorylation ↓ DNMT1	[68]
A549 (AC), H1299 (AC), SK-MES1 (SCC)	2.5–5 mM met alone or in combination with ionizing radiation for 72 h A549 ~ IC_50_ = 75 μM H1299 ~ IC_50_ = 25 μM SK-MES1 ~ IC_50_ = 25 μM	↓ proliferation ↑ radio-sensitization	↑ ATM-AMPK-P53 pathway activity ↓ Akt, mTOR, 4EBP1 pathways ↑ G_1_ cell cycle arrest ↑ apoptosis	[69]

NSCLC, Non-small cell lung cancer; SCLC, Small cell lung cancer; AC, Adenocarcinoma; SCC, Squamous cell carcinoma; LCC, Large cell carcinoma; MSH2, MutS homologue 2; EGFR, Epidermal growth factor receptor; TKI, Tyrosine kinase inhibitor; EMT, Epithelial to mesenchymal transition; SNAIL, Zinc finger protein SNAI1; IGF-1R, Insulin-like growth factor 1 receptor; mTOR, Mammalian target of rapamycin; p70S6K, Ribosomal protein S6K kinase beta-1; NRAS, Neuroblastoma RAS viral oncogene homolog; MAPK, Mitogen activated protein kinase; PI3K, Phosphoinositide-3-kinase; Akt, Protein kinase B; AMPK, AMP-activated protein kinase; MMP-2, Matrix metalloproteinase-2; MMP-9, Matrix metallopeptidase-9; NF-κB, Nuclear factor kappa-light chain-enhancer of activated B cells; VEGF, Vascular endothelial growth factor; STAT3, Signal transducer and activator of transcription 3; ROS, Reactive oxygen species; ERCC1, excision repair cross-complementation 1; PDK1, Pyruvate dehydrogenase lipoamide kinase isozyme 1; TGF-β, Transforming growth factor β; MEK, Mitogen-activated protein kinase kinase; ERK, Extracellular regulated kinase; c-FLIP, CASP-8 and FADD-like apoptosis regulator; p62, Nucleosome p62; DNMT1, DNA methyltransferase 1; p53, Tumor protein p53; 4EBP1, Eukaryotic translation initial factor 4E-binding protein 1; IC_50_, half maximal inhibitory concentration.

**Table 3:** Metformin in lung cancer: in vivo animal studies.

Animal Model	Dose and Duration	Findings	Mechanism	Reference
7 week old female Balb/c mice inoculated subcutaneously with A549 or A431 cells	Drinking water +250 mg/kg/day metformin for 21 days	↑ apoptosis ↓ growth of K-ras mutant tumors	↓ Akt levels ↓ mTOR activation	[39]
8 week old LID mice were given 3 weekly injections of NNK (tobacco carcinogen)	Drinking water +5 mg/mL metformin	↓ lung tumorigenesis	IGF-1 independent mechanism ↓ phosphorylation of multiple RTKs	[70]
6 week old nude mice inoculated with HCC827-pSB388 cells	Drinking water +250 mg/kg body weight metformin, 2 days before tumor inoculation—till the mice were sacrificed	↓ growth and distant metastases ↓ IL-6 induced EMT	↓ STAT3 phosphorylation	[71]
4–6 week old female Balb/c mice were injected subcutaneously with H1299 or CALU-3 GEF-R cells	Drinking water +200 mg/mL metformin +150 mg/kg/day gefitinib for 35 days	↓ proliferation ↑ apoptosis	↓ EGFR phosphorylation ↑ MAPK activation	[40]
6 week old female Balb/cA-nu mice were inoculated with PC-9GR or PC-9 cells subcutaneously into the back next to the left forelimb	Drinking water +1 mg/mL metformin alone or in combination with 250 mg/L gefitinib for 30 days	↓ tumor growth in xenografts with TKI-resistant cancer cells	↓ IL-6 secretion and expression ↓ IL-6 signaling action ↓ EMT	[55]
Male SD rats with average weight of 200 g. Maintained under chloral hydrate anesthesia (500 mg/kg)	Gefitinib 200 mg/kg administered orally once/day for 3 days before animals received a single intratracheal administration of bleomycin (5 mg/kg). Gefitnib and metformin (300 mg/kg) was then continued once every 2 days for the following 21 days	↓ exacerbation of bleomycin-induced pulmonary fibrosis by gefitinib	↓ α-actin and COL1A1 ↓ expression of pSMAD2, pSMAD3, pSTAT3, pAKT and ERK1/2 ↓ TGF-β levels and activation	[51]
6–8 week old balb/c nude mice were subcutaneously injected with AS2 cells into the flanks	Drinking water with or without 500 mg/kg metformin with or without 4 mg/kg cisplatin	↓ xenograft growth ↓ cisplatin induced ROS production and autocrine IL-6 secretion	↓ of STAT 3 pathway	[60]
Balb/c nude mice were subcutaneously injected with A549 cells into their right flanks	40 mg/kg/day metformin alone or in combination with 5 mg/kg/day cisplatin	↓ tumor size	↓ Bcl-2 protein levels ↑ Bax protein expression	[53]
7 week old immunodeficient Balb/c female nude mice injected subcutaneously with A549 cells into the right posterior flanks	Drinking water + 400 mg/kg/day body weight metformin + 30 mg/kg/day. Sorafenib for 40 days. Given either agent alone or in combination.	↓ proliferation	↑ apoptosis phosphorylation of AMPK ↑ inhibition of downstream mTOR signaling	[58]
4–6 week old nude mice bearing H1299 or H1975 cells that were grown subcutaneously	Drinking water +200 mg/mL metformin +25 mg/kg selumetinib for 35 days	Expression changes of mesenchymal proteins, SNAIL and vimentin ↓ tumor metastatic behavior	↓ production of MMP-2 and MMP-9 by reducing NF-κB	[59]
5 week old balb/c-nude mice were subcutaneously injected with A549 or H1299 cells into the right flank	Drinking water +300 mg/kg body weight per day metformin till euthanasia. Xenografts were subjected to 0 Gy or 10 Gy IR while under gaseous anesthesia	↓ of xenograft growth	↑ ATM-AMPK-P53 pathway ↓ Akt-mTOR-4EBP1 pathways in tumors ↓ angiogenesis ↑ expression of apoptosis markers	[69]

K-Ras, V-Ki-ras2 Kirsten rat sarcoma viral oncogene homolog; Akt, Protein kinase B; mTOR, Mammalian target of rapamycin; IGF-1, Insulin-like growth factor 1; RTK, Receptor tyrosine kinase; IL-6, Interleukin-6; EMT, Epithelial to mesenchymal transition; STAT3, Signal transduced and activator of transcription 3; EGFR, Epidermal growth factor receptor; MAPK, Mitogen activated protein kinase; TKI, Tyrosine kinase inhibitor; COL1A1, Collagen type 1 alpha 1; SMAD2, SMAD family member 2; SMAD3, SMAD family member 3; ERK, Extracellular regulated kinase; TGF-β, Transforming growth factor β; ROS, Reactive oxygen species; Bcl-2, B-cell lymphoma 2; Bax, Bcl-2-like protein 4; AMPK, AMP-activated protein kinase; SNAIL, Zinc finger protein SNAI1; MMP-2, Matrix metalloproteinase-2; MMP-9, Matrix metallopeptidase-9; NF-κB, Nuclear factor kappa-light chain-enhancer of activated B cells; p53, Tumor protein p53; 4EBP1, Eukaryotic translation initial factor 4E-binding protein 1.
